# Nanobubbles activate anaerobic growth and metabolism of *Pseudomonas aeruginosa*

**DOI:** 10.1038/s41598-021-96503-4

**Published:** 2021-08-19

**Authors:** Miu Ito, Yuichi Sugai

**Affiliations:** 1 Department of Earth Resources Engineering, Graduate School of Engineering, Kyushu University, 744, Motooka, Nishiku, Fukuoka, 8190395 Japan; 2 Department of Earth Resources Engineering, Faculty of Engineering, Kyushu University, 744, Motooka, Nishiku, Fukuoka, 8190395 Japan

**Keywords:** Bacterial techniques and applications, Microbiology techniques

## Abstract

The effect of nanobubbles on anaerobic growth and metabolism of *Pseudomonas aeruginosa* was investigated. *P*. *aeruginosa* grew earlier in the culture medium containing nanobubbles and the bacterial cell concentration in that culture medium was increased a few times higher compared to the medium without nanobubbles under anaerobic condition. Both gas and protein, which are the metabolites of *P*. *aeruginosa*, were remarkably produced in the culture medium containing nanobubbles whereas those metabolites were little detected in the medium without nanobubbles, indicating nanobubbles activated anaerobic growth and metabolism of *P*. *aeruginosa*. The carbon dioxide nanobubbles came to be positively charged by adsorbing cations and delivered ferrous ions, one of the trace essential elements for bacterial growth, to the microbial cells, which activated the growth and metabolism of *P*. *aeruginosa*. The oxygen nanobubbles activated the activities of *P*. *aeruginosa* as an oxygen source.

## Introduction

*Pseudomonas aeruginosa* is widely distributed in the natural environment, and they can grow under aerobic conditions by respiration with oxygen, while they can also grow under anaerobic conditions by denitrification^1,2^.

Meanwhile, *P*. *aeruginosa* has been found to be capable of producing rhamnolipids, a type of biosurfactant^3–6^, or degrading petroleum hydrocarbons^7–10^. Therefore, a number of studies have reported the use of *P*. *aeruginosa* for bioremediation of soils and water contaminated by petroleum hydrocarbons and heavy metals.

Also, *P*. *aeruginosa* has received attention in the field of petroleum development, which is research area of the authors of this paper. In general, more than half of the crude oil present in underground oil reservoir cannot be recovered by the existing techniques, and the enhanced oil recovery techniques are being studied as new technologies to recover the crude oil remaining in oil reservoir. Microbial enhanced oil recovery technique utilizing effective microorganisms is one of the enhanced oil recovery techniques. The effective microorganism(s) is injected into oil reservoir with their nutrients and cultivated in oil reservoir in the microbial enhanced oil recovery technique. The microbial metabolites such as surfactant, polymer, gases and acids improve the mobility of crude oil in porous system of oil reservoir and bring enhancement of oil recovery. Since the biosurfactant metabolized by *P*. *aeruginosa* is helpful in improving oil recovery, studies on microbial enhanced oil recovery techniques using *P*. *aeruginosa* have been reported^11–16^.

When *P*. *aeruginosa* is used for bioremediation and enhanced oil recovery, it will not be always applied to the aerobic environment. In the enhanced oil recovery, the microorganisms must be exposed to anaerobic environment because depth of oil reservoir is generally more than 1000 m below the surface. In the bioremediation of soil and water, the microorganisms may be exposed to microaerobic or anaerobic environment depending on the depth from the soil or water surface. Bioproduction of rhamnolipid and microbial hydrocarbon degradation, which are useful for bioremediation and enhanced oil recovery, can be more easily proceeded under aerobic conditions^17–19^, so that the growth and metabolic activity of *P*. *aeruginosa* must be activated even under anaerobic conditions to increase the efficacy of the objective in these microbial utilization processes. For this purpose, chemical nutrients that activate the growth and metabolism of the microorganism may be applied to the target field. The environmental impact of those chemical nutrients should be however taken into account because those microbial processes are applied to natural environment. It is also important to minimize the use of those chemical nutrients because target field is usually huge and large amount of nutrients must be prepared for the microbial processes. Therefore, we focused on the potential of nanobubbles for the growth and metabolic activation of *P*. *aeruginosa* under anaerobic conditions in this study. Because nanobubbles can be generated from gas, both the environmental impact and cost are smaller than the chemical nutrients.

Nanobubbles have received attention in various fields in recent years. In the biology field, nanobubbles have been reported to be effective in promoting the growth of plants^20–23^ and shellfish^24,25^. It is suggested that nanobubbles play a role as an oxygen source for those lives by several papers^26–31^. Some papers suggest that the hydroxyl radicals which are generated when the oxygen nanobubbles collapse improve the seed germination percentage^20,32–34^. On the other hand, in the microbiology field, though many studies on the bactericidal effect using nanobubbles such as ozone nanobubbles^35–38^, free radicals generated from nanobubbles^39^, and shock waves emitted by implosion of nanobubbles irradiated with laser light^40^ have been reported, there are only a few examples of examinations for the purpose of promoting the growth of microorganisms^41,42^. Guo et al. investigated the effects of nanobubbles on the growth of *Lactobacillus acidophilus* and its lactic acid production^42^, although the bacterial species was different from our target species. Their studies indicated that the presence of nanobubbles in culture medium of *L*. *acidophilus*, albeit not very large, activated its growth and lactate production during the lag phase and logarithmic phase of *L*. *acidophilus*. They also suggested that nanobubbles altered the zeta potential and concentration of the medium, which in turn activated the growth of *L*. *acidophilus*. They also proposed that a hypothetically negatively charged nanobubbles might be responsible for adsorbing cations in the medium and transporting them to microbial cells.

Even nanobubbles composed of gases other than ozone may inactivate microorganisms due to the generation of free radicals and shock waves generated during the burst of nanobubbles, as mentioned above. However, Yasui et al. reported that those free radicals are generated when physical stimulation is applied to the nanobubbles^43,44^. Also, the shock waves are generated only when the nanobubbles are irradiated with laser light. That is, in situations where no physical stimulation is applied to the nanobubbles, such as in static culture, there is little possibility that the microorganisms will be inactivated by the free radicals, but rather they may be activated.

Therefore, this study investigated the potential of nanobubbles as an activator of the growth and metabolisms of *P*. *aeruginosa*. The gas identity in the nanobubbles was assumed to be an influential factor for the potential of nanobubbles, therefore, this study selected two types of gases such as oxygen and carbon dioxide and investigated the potential of nanobubbles comprising them. As described above, the oxygen nanobubbles can be expected to be an oxygen source for lives. The culture medium with oxygen nanobubbles was used for the culture experiments because of our hypothesis that the oxygen nanobubbles could be oxygen source for *P*. *aeruginosa*, which can grow better under aerobic condition. The carbon dioxide nanobubbles medium was used as the control experiments that didn’t contain oxygen in order to test the hypothesis. It was important to remove solved oxygen completely from the culture medium of the control experiment, carbon dioxide whose solubility was quite higher than that of oxygen was selected in this study. Also, the carbon dioxide nanobubble medium could be used for determining the effect of nanobubble itself for the anaerobic growth and metabolism of *P*. *aeruginosa*. Because the objective of this study is to determine the effect of nanobubble for the anaerobic growth and metabolism of *P*. *aeruginosa*, both culture media with and without nanobubble were prepared. The nanobubbles-free medium was also used as the control experiments that didn’t contain nanobubbles in order to examine the effect of nanobubbles themselves in this study. This study also tried to clarify the mechanisms of activation for the growth and metabolisms of *P*. *aeruginosa* in both nanobubbles media.

## Results and discussion

### Investigation of sterilization methods for nanobubbles water

The method sterilizing the water containing oxygen nanobubbles was examined first of all. The sterilized oxygen nanobubbles water was used for preparing the culture medium in which *P*. *aeruginosa* was purely cultivated.

First, oxygen nanobubbles water that was prepared as described in the Methods section was used to prepare the Cooper’s medium^45^ that is also described in the Methods section. The oxygen nanobubbles medium was autoclaved at 121 °C for 20 min, resulting in the precipitation was observed in that medium while the precipitation was not observed in the nanobubbles-free medium which was autoclaved at the same conditions. These results suggested that some chemical reactions might be occurred in the oxygen nanobubbles medium at high temperature and pressure conditions. The autoclave sterilization was therefore evaluated to be inappropriate method for sterilizing the Cooper’s medium containing nanobubbles in this study. Then, the filtration sterilization was tested as an alternative method sterilizing the nanobubbles medium.

In general, the pore size of the membrane filter used in the filtration sterilization of the medium is 0.22 μm. However, the diameter of the nanobubbles generated by the nanobubble generator used in this study ranged from 50 to 300 nm, suggesting that many nanobubbles cannot pass through the filter and be removed when sterilized the nanobubbles medium using the membrane filter with a pore size of 0.22 μm. On the other hand, when the pore size of the membrane filter is large, the culture medium cannot be completely sterilized because bacteria inhabiting the medium cannot be filtered and removed completely. The pore size suitable for passing the nanobubbles and removing the bacteria was therefore examined in this study.

**Figure 1:**
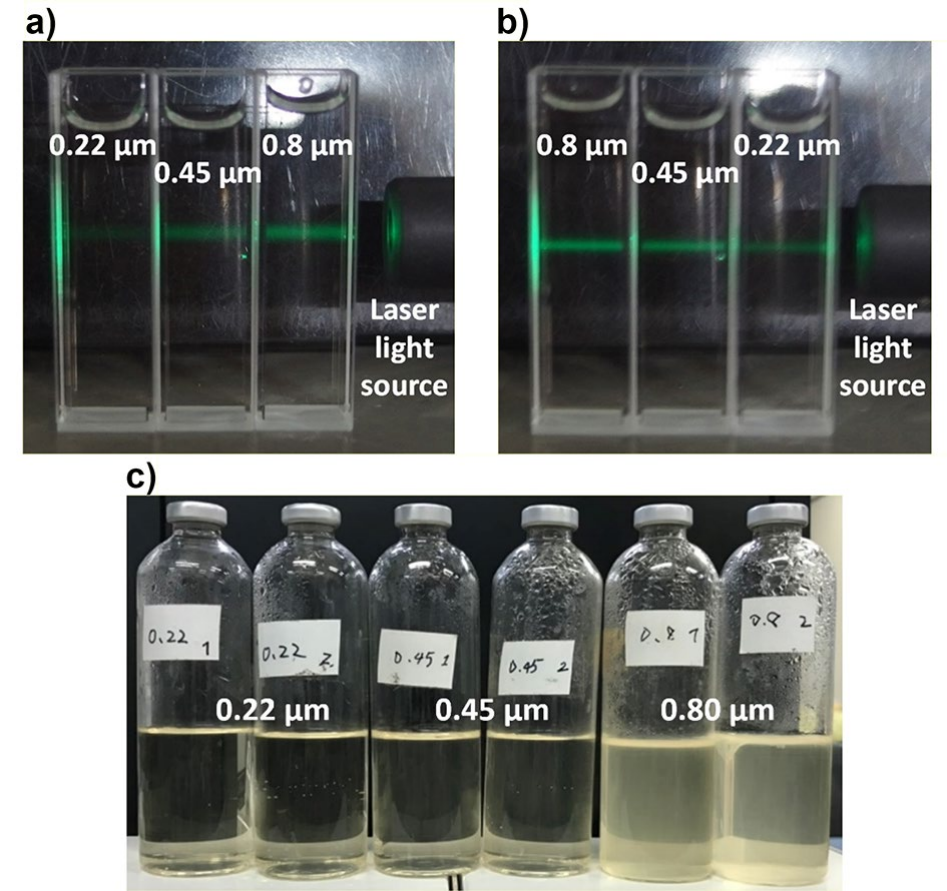
Photo images of scattered light observed by irradiating green laser in the carbon dioxide nanobubbles medium which had been filtered with membrane filters and the culture media which were filtered with membrane filters and anaerobically cultured at 30 °C for 10 days. (**a,b**) The carbon dioxide nanobubbles medium was filtered with membrane filters whose pore size was 0.22 μm, 0.45 μm and 0.80 μm respectively. The green laser light irradiated the carbon dioxide nanobubbles medium from right to left, while the alignment of the samples was opposite each other in (**a,b**). It was clearly observed that the intensity of scattered light became lower as the pore size of filter which had been used for the filtration of the carbon dioxide nanobubbles medium became smaller, suggesting that more nanobubbles had been caught with the filter whose pore size was smaller. (**c**) Each two culture medium on the left, center and right had been filtered with 0.22 μm, 0.45 μm and 0.80 μm respectively. Every culture medium did not contain *P*. *aeruginosa* and the nanobubbles. The head space in the vial bottles were replaced with pure nitrogen. After 10 days cultivation, the culture media which had been filtered with 0.80 μm membrane filters were cloudy with bacteria whereas the culture media filtered with 0.22 μm and 0.45 μm were still remain clear, suggesting the indigenous bacteria had been completely sterilized with membrane filters whose pore size was less than 0.45 μm.

The photographs of carbon dioxide nanobubbles medium that was filtered with 0.22 μm, 0.45 μm and 0.8 μm membrane filters, respectively, and irradiated with green laser in the filtrate are shown in Fig. 1a,b. Since nanobubbles scatter light due to refraction and reflection of light at the gas–liquid interface, scattered light is observed when short-wavelength laser light such as green laser is irradiated on the nanobubbles water, and some of the approximate nanobubbles can be evaluated from the intensity^46^. According to the Fig. 1a,b, the lightest scattered light was observed in the nanobubbles water filtered by a membrane filter with a pore size of 0.8 μm, and the brightness tended to weaken as the pore size decreased. This result suggested that the nanobubbles were filtered and removed from the filtrate by the membrane filter with a small pore size.

Figure 1c shows the nutrient broth medium used for pre-cultivation of *P*. *aeruginosa* described in the “Methods” section after 10 days of cultivation at 30 °C on the medium filtered through the three membrane filters described above. The turbidity of the culture medium was observed in the culture medium filtered by a membrane filter with a pore size of 0.8 μm, and a large number of microorganisms were also observed in the culture medium by the phase contrast microscope observation. On the other hand, no turbidity of the culture medium was observed even after a lapse of 10 days for the medium filtered by a membrane filter with a pore size of 0.45 μm or less as shown in the Fig. 1c, and the microorganisms were not found even by the observation using a phase contrast microscope. It was shown that the successful sterilization in the culture medium was properly sterilized by using those membrane filters.

The nanobubble number density in the oxygen nanobubbles water and the carbon dioxide nanobubbles water which were filtered with the membrane filter with a pore size of 0.45 μm was 4.0 × 10^6^/mL and 1.0 × 10^7^/mL respectively. It was assumed that more nanobubbles were generated in the carbon dioxide dissolved water because the solubility of carbon dioxide is higher than that of oxygen^47^. The median diameter of the oxygen nanobubble and carbon dioxide nanobubble was 119.6 nm and 128.6 nm respectively. It should be noted that the nanoparticles were also detected in the nanobubbles-free water which was filtered with the same membrane filter with the density of 2.2 × 10^7^/mL. The above nanobubble number density was therefore calculated by deducting the nanoparticle number density in the nanobubbles-free water from that in the nanobubbles water.

From the above results, it was shown that a sterile medium containing nanobubbles was produced by using a membrane filter with a pore size of 0.45 μm. The culture medium therefore prepared by this method was to be used in the following experiments. It should be noted that no microorganisms were detected in the non-inoculated culture media in all the experiments and the repeatability of the result that the successful sterilization in the culture medium was properly sterilized by using the membrane filter with pore size of 0.45 μm was supported.

### Cultivation experiments of *P*. *aeruginosa* with nanobubbles-containing culture medium

**Figure 2:**
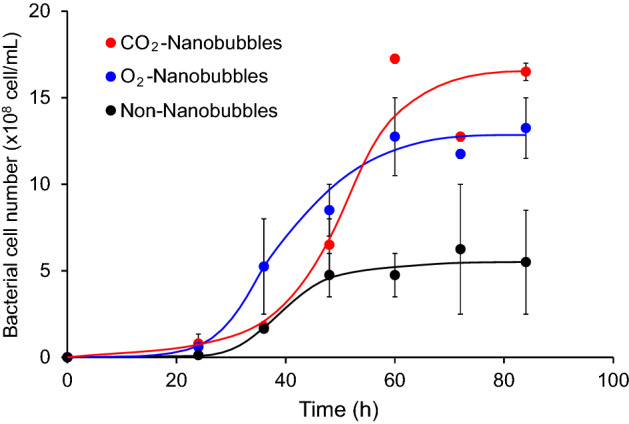
Growth curve of *P*. *aeruginosa* cultured in Cooper’s medium. Bacterial cell number of *P*. *aeruginosa* cultured in the carbon dioxide nanobubbles medium (red closed circles), oxygen nanobubbles medium (blue closed circle) and nanobubbles-free medium (black closed circle).

Figure 2 shows the growth curves of *P*. *aeruginosa* in the nanobubbles-free medium, oxygen nanobubbles medium and carbon dioxide nanobubbles medium. In all culture media, the lag phase was observed in which no growth of *P*. *aeruginosa* was observed for 24 h from the start of the cultivation, followed by a logarithmic phase of rapid growth. The transition of the growth phase to the logarithmic phase was found in the oxygen nanobubbles medium after 24 h from the start of the cultivation. The bacterial cell concentration reached 1.2 × 10^9^ cells/mL at 60 h from the start of the cultivation in the oxygen nanobubbles medium. On the other hand, the bacterial cell concentration in the carbon dioxide nanobubbles medium remained similar to that in the nanobubbles-free medium until 36 h after the start of the cultivation. The logarithmic growth was observed afterwards, and the bacterial cell concentration reached 1.7 × 10^9^ cells/mL at 84 h in the carbon dioxide nanobubbles medium. The bacterial cell concentration in the nanobubbles-free medium remained at 5.5 × 10^8^ cells/mL, which was less than half of the bacterial cell concentration at the 84-h time point in the medium with nanobubbles mentioned above. These results obviously suggest that the growth of *P*. *aeruginosa* is activated under the presence of nanobubbles. The growth of *P*. *aeruginosa* in the carbon dioxide nanobubbles medium was a little later than that in the oxygen nanobubbles medium, however, the final bacterial cell concentration in the carbon dioxide nanobubbles medium was higher than that in the oxygen nanobubbles medium. Because initial pH of the carbon dioxide nanobubbles medium was 6.6 which was a little lower than the initial pH of the oxygen nanobubbles medium, which was 6.8, the growth of *P*. *aeruginosa* was affected in its early culture stage in the carbon dioxide nanobubbles medium.

**Figure 3:**
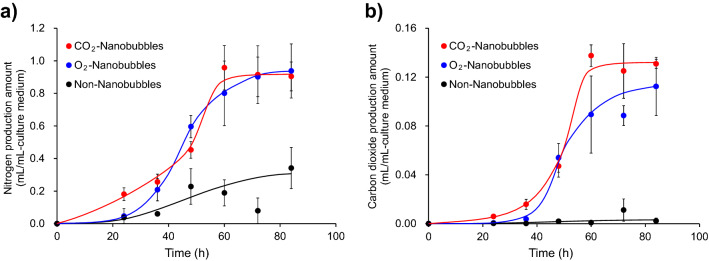
Amount of gas generated by the cultivation of *P*. *aeruginosa* in the Cooper’s medium. (**a**) Nitrogen production amount in the carbon dioxide nanobubbles medium (red closed circle), oxygen nanobubbles medium (blue closed circle) and nanobubbles-free medium (black closed circle). (**b**) Carbon dioxide production amount in the carbon dioxide nanobubbles medium (red closed circle), oxygen nanobubbles medium (blue closed circle) and nanobubbles-free medium (black closed circle).

The gas production by *P*. *aeruginosa* was observed in the cultivation experiments of this study. According to our gas analysis, *P*. *aeruginosa* produced both nitrogen and carbon dioxide which were metabolites related to denitrification and anaerobic fermentation of glucose respectively. Figure 3 shows production of nitrogen and carbon dioxide in the experiments cultivating *P*. *aeruginosa* in each culture medium. Both nitrogen and carbon dioxide began to be generated from just after starting the cultivation in the carbon dioxide nanobubbles medium. The gas generation in the oxygen nanobubbles medium was observed a little later than that in the carbon dioxide nanobubbles medium. Nitrogen production amount in the nanobubbles-free medium was one-third and of that in both nanobubbles media. Carbon dioxide was little generated in the nanobubbles-free medium. These results suggest that the nanobubbles activates gas generation of *P*. *aeruginosa*. It should be noted that the volume of carbon dioxide produced from the carbon dioxide nanobubbles medium into which P. aeruginosa had not been inoculated was less than 0.01 mL/mL-culture medium. More than 90% of carbon dioxide detected in this study can be therefore assumed to be produced by the fermentation.

**Figure 4:**
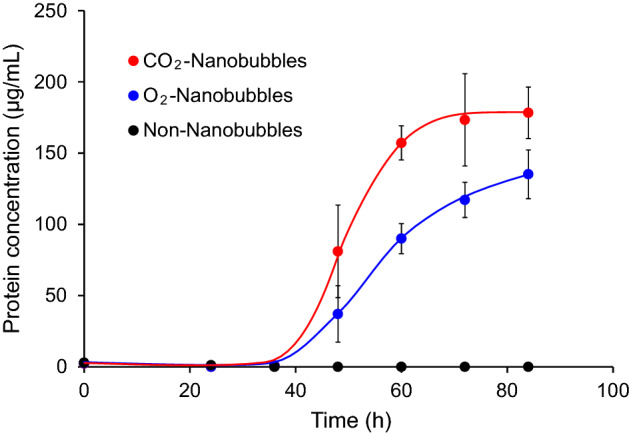
Concentration of protein generated by the cultivation of *P*. *aeruginosa* in the Cooper’s medium. Protein concentration in the carbon dioxide nanobubbles medium (red closed circle), oxygen nanobubbles medium (blue closed circle) and nanobubbles-free medium (black closed circle).

*Pseudomonas aeruginosa* has been reported to metabolize heme iron acquisition protein (HasA) as one of the mechanisms of iron acquisition essential for its growth^48–52^. This led us to investigate the production of proteins to evaluate the metabolic activity of *P*. *aeruginosa* in each culture medium in this study. The total protein concentration was determined by the method described in the “Methods” section in this study. Figure 4 shows the time course of the total protein concentration in the culture. The protein production was observed only in the oxygen nanobubbles media and carbon dioxide nanobubbles media 36 h after the start of the cultivation. The proteins were produced most actively between 36 and 60 h after the start of the cultivation, and proteins were continuously produced thereafter until the 84 h elapsed. The highest total protein concentration was detected, especially in the carbon dioxide nanobubbles medium, which was about 1.3 times the concentration of total protein in the oxygen nanobubbles medium at 84 h after the start of the cultivation.

**Figure 5:**
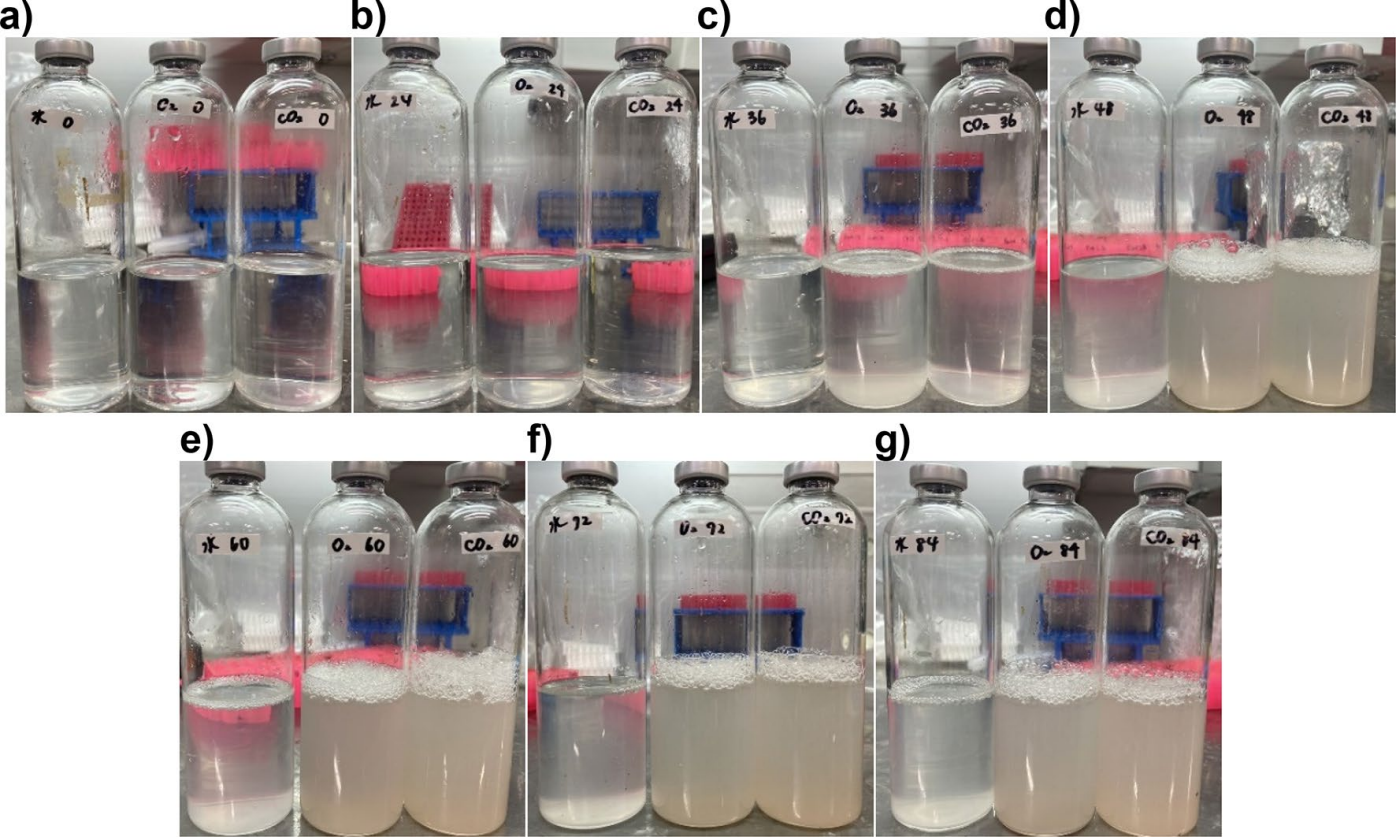
Photo images of culture solution of *P*. *aeruginosa* in the Cooper’s medium. Every photo image shows culture solution of *P*. *aeruginosa* cultured in from the left, nanobubbles-free medium, oxygen nanobubbles medium and carbon dioxide nanobubbles medium. (**a**) Culture solution just after the start of cultivation. (**b**) Culture solution after 24 h cultivation. (**c**) Culture solution after 36 h cultivation. (**d**) Culture solution after 48 h cultivation. (**e**) Culture solution after 60 h cultivation. (**f**) Culture solution after 72 h cultivation. (**g**) Culture solution after 84 h cultivation.

Finally, photographs of the culture solution of *P*. *aeruginosa* at each elapsed time from the start of the cultivation are shown in Fig. 5. The amount of foam produced on the surface of the culture solution was overwhelmingly high in the culture media containing nanobubbles, indicating active growth and metabolism of *P*. *aeruginosa*. The time of bubble formation was generally consistent with the time of increased protein levels described above, suggesting that bubbles were produced by the proteins and gases produced by *P*. *aeruginosa*.

### Investigation of the effect of nanobubble number density on the growth and metabolism of *P*. *aeruginosa*

**Figure 6:**
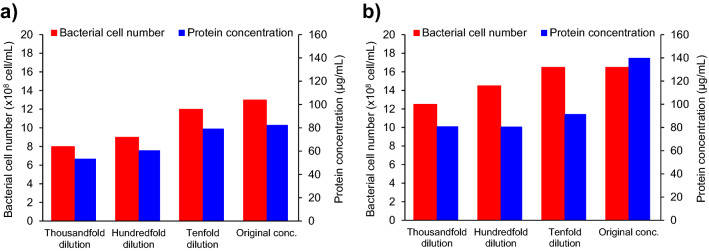
Bacterial cell number and protein concentration after 84 h cultivation of *P*. *aeruginosa* in the nanobubbles media. (**a**) Bacterial cell number (red bars, set on the left-hand axis) and protein concentration (blue bars, set on the right-hand axis) detected in the oxygen nanobubbles medium after 84 h cultivation. (**b**) Bacterial cell number (red bars, set on the left-hand axis) and protein concentration (blue bars, set on the right-hand axis) detected in the carbon dioxide nanobubbles medium after 84 h cultivation.

The cultivation experiments of *P*. *aeruginosa* were carried out using culture media containing nanobubbles with different density in order to confirm the potential of nanobubbles for the growth and metabolic activation of *P*. *aeruginosa* under anaerobic conditions. The nanobubble number density in the Cooper’s medium was adjusted by diluting the original nanobubbles water with tap water which was bubbled with each gas. Figure 6 shows the bacterial cell concentration and protein concentration in the oxygen nanobubbles medium and the carbon dioxide nanobubbles medium which were cultivated for 84 h. The number density of both oxygen nanobubbles and carbon dioxide nanobubbles caused differences in both bacterial cell concentration and protein production, and the higher the nanobubble number density, the higher those values as shown in the figure. The experimental results that the degree of microbial activation depended on the nanobubble number density strongly support the microbial activation effect of nanobubbles.

### Investigation of the role of nanobubbles activating the growth and metabolism of *P*. *aeruginosa*

It was clearly shown that both oxygen nanobubbles and the carbon dioxide nanobubbles activated the growth and metabolism of *P*. *aeruginosa*. Rather, a trend toward more activation of the growth and metabolism of *P*. *aeruginosa* was observed when nanobubbles containing carbon dioxide as a major gas, suggesting that nanobubbles played not only a role as a source of oxygen to *P*. *aeruginosa* but also other roles. Guo et al. investigated the effects of nanobubbles on the growth of *Lactobacillus acidophilus* and its lactate production activity. Their study built a hypothesis that the transfer of negatively charged nanobubbles to microbial cells by adsorbing cations in the medium leads to more efficient delivery of cations to microbial cells than simply by diffusing cations to microbial cells, thus enhancing *L*. *acidophilus* growth and lactate production activity. Because we also hypothesized that the similar mechanism might be applied to the case of *P*. *aeruginosa*, the zeta potential of both oxygen nanobubbles and carbon dioxide nanobubbles in tap water and the Cooper’s medium were measured to understand the surface charge of those nanobubbles in this study.

The zeta potentials of oxygen nanobubbles in the tap water and the Cooper’s medium were not so much different such as − 14.66 mV and − 18.16 mV respectively. On the other hand, the zeta potentials of carbon dioxide nanobubbles in the tap water and the Cooper’s medium were quite different such as − 18.07 mV and 2.98 mV respectively. Based on those results, we discussed the activation mechanisms caused by the carbon dioxide nanobubbles and the oxygen nanobubbles as follows.

The carbon dioxide nanobubbles, which had negative zeta potential value in tap water, came to have positive zeta potential value in the Cooper’s medium. That is, cations in Cooper’s medium were attracted on the negatively charged carbon dioxide nanobubbles and changed to positive charges. It seems that the carbon dioxide nanobubbles which became positively charged by attracting the cations became accessible to the negatively charged microbial cells, and the supply of cations to *P*. *aeruginosa* was promoted, and the growth and metabolism of *P*. *aeruginosa* were activated. The Cooper’s medium contains sodium ion, potassium ion, ammonium ion, magnesium ion, calcium ion, ferrous ion and manganese ion as cations as indicated in the Methods section. Of these, ferrous ion and manganese ion are generally classified as trace essential elements to microorganisms. Because concentration of those cations was quite lower than that of other cations in the Cooper’s medium, microorganisms may be sensitive to the fractional change in concentration of those trace essential elements. We therefore focused on those trace essential elements and investigated the influences of those elements on the growth and metabolism of *P*. *aeruginosa* with and without the carbon dioxide nanobubbles.

**Figure 7:**
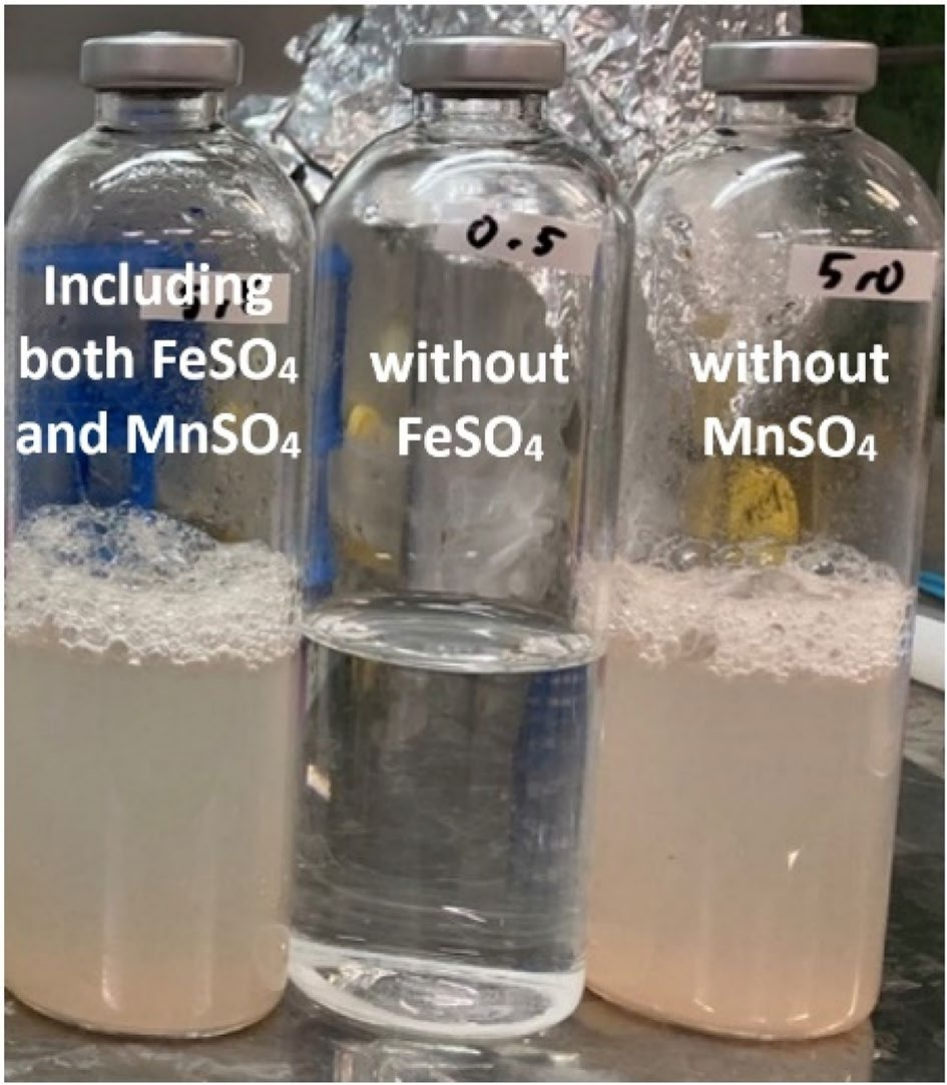
Photo image of the culture solution of *P*. *aeruginosa* cultured in the Cooper’s medium for 2 days. *P*. *aeruginosa* was cultured in the Cooper’s medium containing both ferrous sulfate and manganese sulfate (left), without ferrous sulfate (center) and without manganese sulfate (right). Both turbidity in the culture solution and foam, which were derived by the growth and metabolism of *P*. *aeruginosa* were observed in only culture solution containing ferrous sulfate, suggesting ferrous ion is a trace element which governs the growth of the *P*. *aeruginosa*.

First, in order to clarify the effect of ferrous ion and manganese ion on the growth of *P*. *aeruginosa*, each of the amounts added was 5 times that of Cooper’s medium, and culture experiments of *P*. *aeruginosa* were performed using a medium containing both cations and a medium containing only one of them. The nanobubbles were not contained in every medium in order to evaluate only the effect of those cations on the growth of *P*. *aeruginosa* in this experiment. Consequently, the growth of *P*. *aeruginosa* without or with manganese ion was observed in the medium in which ferrous ion was added 5 times the ferrous ion concentration in Cooper’s medium as shown in Fig. 7. On the other hand, in the medium in which manganese ion was added in 5 times of the manganese ion concentration in Cooper’s medium, a difference in the growth of *P*. *aeruginosa* was observed depending on the presence or absence of ferrous ion. These results indicate that ferrous ion is a trace element which governs the growth of the *P*. *aeruginosa*.

**Figure 8:**
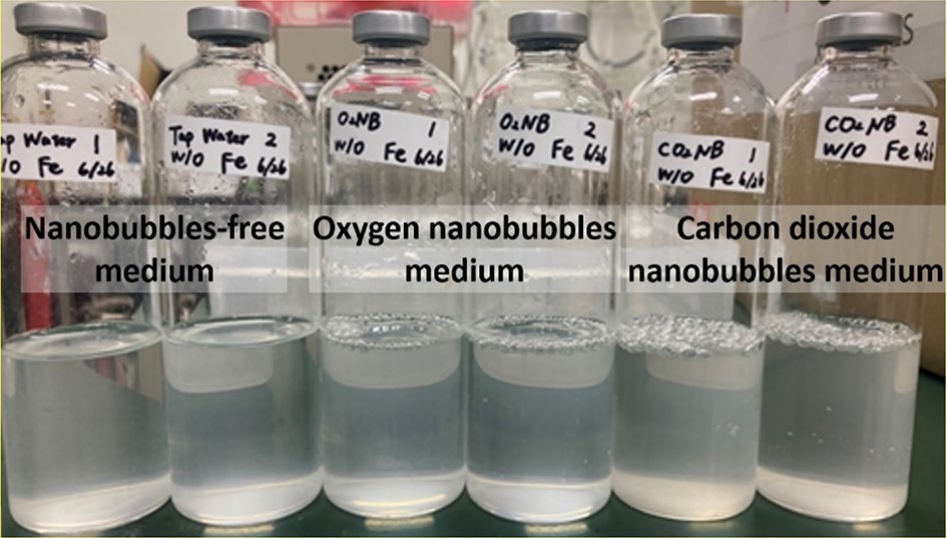
Photo image of the culture solution of *P*. *aeruginosa* cultured in the Cooper’s medium without addition of ferrous sulfate for 3 days. After 3 days cultivation, the foam generation by *P*. *aeruginosa* was observed on the surface of the culture solution which contained the oxygen nanobubbles and the carbon dioxide nanobubbles whereas it was not observed on the surface of the culture solution which did not contain the nanobubbles. The foam was the most obviously generated in the carbon dioxide nanobubbles medium, suggesting the carbon dioxide nanobubbles has action for activating *P*. *aeruginosa* even under low ferrous ion concentration condition.

Next, we prepared the nanobubbles-free medium, the oxygen nanobubbles medium and the carbon dioxide nanobubbles medium without adding ferrous ion, and inoculated *P*. *aeruginosa* into those mediums to conduct the cultivation experiments validating the above hypothesis. The results are shown in Fig. 8. The foam which was generated by *P*. *aeruginosa* was obviously observed on the surface of the culture medium containing the nanobubbles after 3 days cultivation, whereas the foam was not observed in the nanobubbles-free medium. This result suggests that the metabolism of *P*. *aeruginosa* was more active in the medium containing the nanobubbles than that in the nanobubbles-free medium. Because the concentration of ferrous ion in tap water of Japan is very low such as less than 50 μg/L^53^, *P*. *aeruginosa* had been assumed not to be so active in the nanobubbles-free medium. The foam generation by *P*. *aeruginosa* was the most obviously found in the culture medium containing the carbon dioxide nanobubbles.

**Figure 9:**
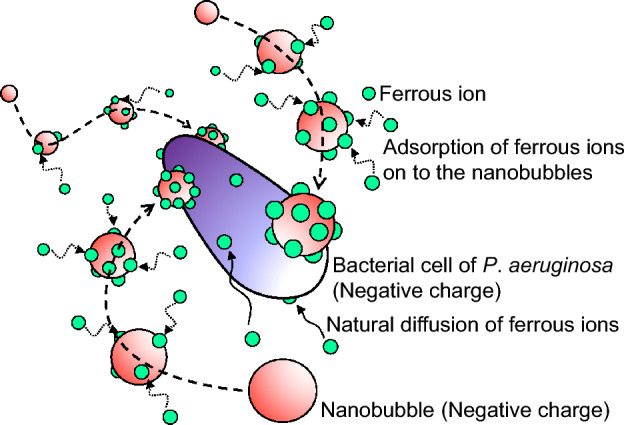
Schematic image of mechanism of ferrous ions delivery to microbial cells by the carbon dioxide nanobubbles. Because the carbon dioxide nanobubbles (red spheres) are negatively charged, cations including ferrous ions (green spheres) are adsorbed on to the surface of the carbon dioxide nanobubbles and change to positive charges. Because bacterial cells (purple rod) are also negatively charged, the carbon dioxide nanobubbles on which ferrous ions are adsorbed are accessible to the bacterial cells and ferrous ions are delivered to the bacteria. Ferrous ions are also delivered to bacterial cells due to natural diffusion, however, the number of ferrous ions delivered to bacterial cells by the carbon dioxide nanobubbles is larger than that delivered due to natural diffusion.

This experimental result supports the hypothesis proposed by Guo et al. That is, the nanobubbles adsorbed the ferrous ions and deliver them to the microbial cells in the nanobubbles-medium as shown in Fig. 9. If the concentration of ferrous ion is low in the nanobubbles-free medium, ferrous ions cannot be enough delivered to the microbial cells by only natural diffusion. Even if the concentration of ferrous ion is low in the nanobubbles medium, ferrous ions can be enough delivered to the microbial cell by the nanobubbles and *P*. *aeruginosa* can actively grow in the nanobubbles medium. In addition, it is expected that the higher nanobubble number density, the greater amount of ferrous ions transported, which adequately explains the experimental results that the degree of microbial activation depends on the nanobubble number density.

On the other hand, the zeta potential of the oxygen nanobubbles was negative in both tap water and Cooper’s medium. Therefore, it cannot reasonably be assumed that the negatively charged oxygen nanobubbles became accessible to the negatively charged microbial cells. Since oxygen was bubbled into water when the oxygen nanobubbles water was prepared, the initial DO concentration of the oxygen nanobubbles medium was the highest in the three kind of media as 9.0 mg/L. The initial DO concentration of culture medium might affect the growth of *P*. *aeruginosa* which grows better under aerobic condition than anaerobic condition. Moreover, the oxygen nanobubbles themselves also served as oxygen sources to *P*. *aeruginosa*, therefore, oxygen condition in the oxygen nanobubbles medium was the most favorable for *P*. *aeruginosa* among in the three media. *P*. *aeruginosa* therefore might have been more vigorous in growth and metabolism in the oxygen nanobubbles medium than nanobubbles-free medium whose oxygen condition was worse. In addition, it is expected that the higher nanobubble number density, the greater amount of oxygen supplied, which adequately explains the experimental results that the degree of microbial activation depends on the nanobubble number density.

Thus, the mechanisms by which *P*. *aeruginosa* was activated in the carbon dioxide nanobubbles medium and the oxygen nanobubbles medium are different.

## Conclusions

The effect of nanobubbles on the growth and metabolic activity of *P*. *aeruginosa* was investigated in this study. The results showed that the growth and metabolic activities of *P*. *aeruginosa* were activated by the presence of nanobubbles in the culture medium. This effect was dependent on the gas identity in the nanobubbles, and the carbon dioxide nanobubbles were more effective than the oxygen nanobubbles medium.

The carbon dioxide nanobubbles came to have positive zeta potential value in the Cooper’s medium. That is, cations in Cooper’s medium were attracted on the negatively charged carbon dioxide nanobubbles and changed to positive charges. The carbon dioxide nanobubbles which became positively charged by attracting the cations became accessible to the negatively charged microbial cells, and the supply of cations especially ferrous ions to *P*. *aeruginosa* was promoted, and the growth and metabolism of *P*. *aeruginosa* were activated. On the other hand, *P*. *aeruginosa* was more vigorous in growth and metabolism in the oxygen nanobubbles medium than nanobubbles-free medium because *P*. *aeruginosa* was supplied with oxygen as dissolved oxygen and oxygen nanobubbles.

## Methods

### Preparation of nanobubbles water

Water containing nanobubbles (hereinafter called “nanobubbles water”) was prepared using a commercially available nanobubbles generator (Art Verre Internation Co., Ltd., Minoh, Japan). The nanobubbles generator is tubular, and a polyvinyl chloride cylinder is placed in the tube. A number of small rhombic columnar projections made of silicone rubber are installed in a staggered grid on the surface of the cylinder. When water flows in the nanobubbles generator, water flows while impinging on the protruding surface, and turbulent flow due to the flip-flop phenomenon occurs. This turbulent flow results in the generation of minute vortices in the water that generate bubbles by cavitation. Further bubbles are collapsed and refined by the Coanda effect. The nanoscale bubbles, nanobubbles can be generated in water by that effect^54,55^. The manufacturer has shown that the nanobubbles with bubble diameters ranging from 50 to 200 nm are generated in more than 100 million/mL in the water^56^. In addition, Jadhav et al. and Yang et al. reported that the amount of nanobubbles produced increased when water containing ethanol was used^57,58^, and this study also prepared nanobubbles water using water supplemented with 1% ethanol to increase the nanobubble number density in the nanobubbles water.

Tap water added to a bucket to provide tap water supplemented with 1.1% ethanol was introduced to the nanobubbles generator using a submersible pump. The nanobubbles water discharged through the nanobubbles generator was recovered in a glass beaker, which was used for the preparation of the culture medium. Since the source of the bubbles produced by cavitation described above is the gas dissolved in water, it was considered that the nanobubble number density was also increased by increasing the dissolved amount of gas. The tap water which had been cooled to 14 °C was therefore used as the source water of the nanobubbles water in order to increase the dissolved amount of gas in the source water. Moreover, the source water was bubbled with oxygen or carbon dioxide in order to increase the dissolved amount of each gas in the source water.

Consequently, saturated solubility of oxygen in the normal tap water was 25.2 mg/L at 14 °C and 1 atm, whereas that in the source water which had been bubbled with oxygen at the same temperature was 27.5 mg/L. Also, the saturated solubility of carbon dioxide in the normal tap water was 2.1 g/L at 14 °C and 1 atm, whereas the concentration of dissolved carbon dioxide in the source water which had been bubbled with carbon dioxide was 1.4 g/L at the same temperature. The concentration of oxygen and carbon dioxide dissolved in the source water was equal to or close to the saturated dissolved amount of each gas at that temperature.

The brightness of scattered light observed by irradiating the nanobubbles water with green laser was evaluated using ImageJ, which was an image analysis software. The brightness of scattered light observed in the nanobubbles water which had been bubbled with oxygen at 14 °C and supplemented with 1.1% ethanol was more than three times higher than that observed in the nanobubbles water prepared using the tap water without ethanol and oxygen bubbling. This result suggests that the nanobubble number density was increased by adding ethanol into the source water and bubbling the source water with gas.

In addition, those nanobubbles water was preserved in a sealed condition at 30 °C for 20 days. The brightness of the scattered light was observed similarly after the preservation term. The results showed that the nanobubbles water prepared with the source water supplemented with ethanol and bubbled with oxygen at 14 °C had comparable brightness to that immediately after preparation, suggesting that nanobubbles were stably present in the sample even after 20 days from sample preparation. All of the microbial culture experiments in this study were completed within 20 days, therefore, the microbial culture experiments were conducted in the presence of stable nanobubbles at all times.

The number density and size distribution of nanoparticle in the nanobubbles-free water, oxygen nanobubbles water and carbon dioxide nanobubbles water which were filtered with a membrane filter was determined using Zeta View PMX100 (MicrotracBEL Corp., Osaka, Japan). The zeta potential of the oxygen nanobubbles and the carbon dioxide nanobubbles in tap water and the Cooper’s medium were also measured using the same equipment.

In this paper, we refer to the nanobubbles water prepared by oxygen bubbling and carbon dioxide bubbling as the oxygen nanobubbles water and the carbon dioxide nanobubbles water respectively.

### Bacterial species and culture medium

*Pseudomonas aeruginosa* ATCC10145 was used as the test microorganism for this study. First, that strain was inoculated into a medium containing 4 g/L of Nutrient Broth (Becton, Dickinson and Company, FranklinLakes, NJ, USA) sterilized at 121 °C for 20 min, and the culture solution aerobically cultured at 30 °C for 3 days was used as a preculture solution as an inoculation source in the experiment.

Cooper’s medium was used for the culture experiment in this study. The culture medium was prepared by adding 40 g of glucose, 5.7 g of disodium hydrogen phosphate, 4.0 g of potassium dihydrogen phosphate, 4.0 g of ammonium nitrate, 0.1 g of magnesium sulfate, 8.0 mg of calcium chloride, 1.0 mg of ethylenediamine tetraacetic acid, 0.6 mg of ferrous sulfate, and 0.2 mg of manganese sulfate, respectively, to 1 L of tap water. The culture medium containing nanobubbles was prepared by the following procedure.

First, among the components described above, a concentrate prepared so as to have a concentration of 10 times that of components other than glucose, ferrous sulfate, and manganese sulfate was prepared. 5 mL of the concentrate was poured into 126 mL vials and autoclaved at 121 °C for 20 min. On the other hand, a solution in which glucose, ferrous sulfate and manganese sulfate were added to tap water or nanobubbles water containing 1.1% of ethanol by 1.1 times the amount described above was subjected to filtration sterilization using a gamma sterilized membrane filter having a pore size of 0.45 μm. 45 mL of this filter sterilized solution was added into the vial containing 5 mL of sterile concentrate as previously described.

### Culture experiment

The bacterial cell concentration in the preculture solution was counted by the direct counting method using phase contrast microscopy. Then, the preculture solution was inoculated into Cooper’s medium so that the initial bacterial cell concentration was 5.0 × 10^4^ cells/mL. Thereafter, the vial bottle was sealed with a sterilized butyl rubber cap and an aluminum seal. The gas phase in the vial bottle was completely replaced with pure nitrogen to be an anaerobic condition. The bacteria were cultured at 30 °C.

In this study, 14 vial bottles with the same culture medium were prepared for an experiment. Two vial bottles were randomly sampled after 0 h, 24 h, 36 h, 48 h, 60 h, 72 h and 84 h elapsed from the start of cultivation. Experiments investigating the effects of nanobubbles on the growth and metabolism of the *P*. *aeruginosa* used the nanobubbles-free medium and nanobubbles medium which had been prepared using oxygen nanobubbles water (hereinafter called “oxygen nanobubbles medium”) or carbon dioxide nanobubbles water (hereinafter called “carbon dioxide nanobubbles medium”). In the experiment which examined the mechanisms of the effect of nanobubbles, the culture medium prepared using tap water without nanobubbles or carbon dioxide nanobubbles water with Cooper’s medium without addition of ferrous sulfate and manganese sulfate was used. All culture experiments consistently used non-inoculated culture medium as a negative control and cultured it under the same conditions as the culture medium inoculated with *P*. *aeruginosa*.

The culture solution sampled at each elapsed time was subjected into various measurements such as bacterial cell concentration, gas production amount, gas component, pH of the culture solution and protein concentration of the culture solution.

### Measurements

Fast of all, the gas production amount was measured by the water displacement method. A vial bottle containing the culture solution was placed in water, and a needle was pierced into the butyl rubber cap in the water to collect gas into a graduated cylinder. After the gas volume in the cylinder had been measured, the gas was transferred into a 10 mL vial bottle in water and stored sealed with a butyl rubber cap and screw cap. The components of the gas were measured using a gas chromatograph. A gas chromatograph GC-14B (Shimadzu Corporation, Kyoto, Japan) equipped with both thermal conductivity detector and flame ionization detector and a stainless column filled with Porapak Q (80–100 mesh) was used in this study. The column temperature was 100 °C and the flow rate of helium carrier gas was 30 mL/min. The pH was measured using a pH meter, LAQUA twin pH-22B (HORIBA, Ltd., Kyoto, Japan). Protein concentration of the culture solution was measured using TaKaRa Bradford Protein Assay Kit (Takara Bio Inc., Shiga, Japan) and a spectrophotometer UV-2450 (Shimadzu Corporation, Kyoto, Japan) in accordance with the instruction manual.
